# Selective Serotonin Reuptake Inhibitors (SSRIs), Childhood and Adolescent Depression, and Suicidality Following the FDA's 2004 Black Box Warning: A Systematized Literature Review

**DOI:** 10.7759/cureus.100438

**Published:** 2025-12-30

**Authors:** Harlin Kaur, Aksha Memon

**Affiliations:** 1 Pharmacology, Creighton University School of Medicine, Phoenix, USA

**Keywords:** adolescents, cognitive behavioral therapy, fda black box warning, major depressive disorder, selective serotonin reuptake inhibitor, suicidality

## Abstract

Major depressive disorder (MDD) in adolescents is a significant public health concern. When left untreated, it can lead to poor academic performance, substance use, and a heightened risk of suicide. The U.S. Food and Drug Administration (FDA) issued a black box warning on selective serotonin reuptake inhibitors (SSRIs) for patients under 18 due to concerns about increased suicidality - a decision that remains debated given SSRIs’ role as first-line treatment for depression and the inherent suicide risk of untreated MDD.

This systematized literature review examined the current evidence on the association between SSRI use and suicidality in adolescents with MDD following the FDA black box warning. Following a comprehensive PubMed search, studies were selected using predefined inclusion and exclusion criteria. Eight studies were selected for further data extraction and synthesis.

Overall, observational studies found a small but measurable increase in suicidal risk with SSRI use, particularly during the early treatment phase, highlighting the need for close monitoring. Two randomized controlled trials (RCTs) reported increased treatment-emergent suicidality with SSRI monotherapy compared to cognitive behavioral therapy (CBT) or combination therapy, while two other RCTs found no significant association. Across studies, combination therapy with SSRIs and CBT tended to yield better outcomes and lower rates of suicidality.

Current evidence reveals that SSRI treatment in childhood and adolescent MDD is associated with a small but measurable increase in suicidality, particularly in early treatment, underscoring the need for close clinical monitoring and concurrent psychotherapy. Clinicians are encouraged to monitor closely for suicidality during early treatment and to carefully weigh the benefits of SSRIs against the risks of untreated depression.

## Introduction and background

Major depressive disorder (MDD) in children and adolescents represents a significant public health issue. Data from the Centers for Disease Control and Prevention (CDC) show that approximately 4% of individuals under 18 years have been formally diagnosed with MDD, while 18% report depressive symptoms [1]. The rising prevalence of MDD among adolescents over the past decade has also been associated with increased engagement on various online social networking platforms, such as TikTok, Snapchat, and Instagram, which frequently expose them to social comparisons with unfamiliar peers, thereby fostering feelings of inadequacy and diminished self-worth [2]. Compared to adults, adolescents with MDD are undertreated and underdiagnosed, possibly due to their indiscriminate presentation of depressive symptoms, such as irritability, aggressive behavior, and school refusal [3]. Evidence-based guidelines recommend pharmacotherapy, psychotherapy, or a combination of both as first-line treatments for moderate MDD [4-6]. Current guidelines for pharmacotherapy include selective serotonin reuptake inhibitors (SSRIs), selective norepinephrine reuptake inhibitors (SNRIs), tricyclic antidepressants (TCAs), and other atypical antidepressants [6]. The rate of antidepressant prescription and use steadily increased over time for the treatment of adolescent MDD until a landmark black box warning was placed [7]. 

In October 2004, the U.S. Food and Drug Administration (FDA) issued a black box warning regarding the use of SSRIs for the treatment of MDD in patients under 18 years of age, citing concerns about an increased risk of suicidal ideation and behavior [8]. This decision followed the conclusion of the United Kingdom’s Medicines and Healthcare Products Regulatory Agency (MHRA), which reported that, with the exception of fluoxetine, SSRIs demonstrated limited efficacy in randomized clinical trials and were associated with an elevated risk of suicidal behavior in adolescents with MDD. The FDA’s warning has remained controversial, as critics argue that it was based on limited evidence and that the subsequent underutilization of SSRIs other than fluoxetine in pediatric MDD may disproportionately emphasize the potential risks while neglecting the substantial therapeutic benefits of pharmacological treatment [8].

This systematized review of the literature [9] aims to evaluate the association between SSRI use and suicidality in patients younger than 18 years with a clinical diagnosis of MDD. A secondary objective is to provide an updated overview of the tolerability and safety profile of SSRI pharmacotherapy in this population.

Current evidence indicates that children and adolescents with depression are at substantially elevated risk of adverse outcomes, including poor academic achievement, substance use disorders, suicidal ideation and attempts, criminal convictions, early parenthood, physical health problems, premature mortality, and social isolation, even after adjusting for childhood adversity and co-occurring psychiatric disorders [10,11]. Despite these risks, MDD remains underdiagnosed and undertreated in pediatric populations [11].

Following the FDA’s 2004 black box warning, a large cohort study demonstrated a 31% relative reduction in antidepressant use among adolescents [12]. Similar declines were observed across adult populations, despite the advisory targeting only youth at risk of suicidality. This widespread decrease in prescribing suggests both heightened caution among clinicians and potential discouragement among patients regarding SSRI treatment. Collectively, these epidemiological findings underscore the need for greater clarity on the comparative risks, highlighting that the consequences of untreated depression may substantially outweigh the relatively small risk of suicidality associated with SSRI use.

First-line therapy for pediatric MDD includes evidence-based psychotherapeutic interventions, such as cognitive behavioral therapy (CBT) and interpersonal therapy [13]. The only pharmacologic agents currently approved by the U.S. FDA for pediatric MDD are fluoxetine (for ages 8-18) and escitalopram (for ages 12-17) [13]. Among the five major classes of antidepressants, SSRIs are the most frequently prescribed, owing to their comparatively favorable safety, tolerability, and side-effect profiles [14,15]. Common side effects notable for SSRI discontinuation in the initial phase include restlessness, anxiety, and gastrointestinal upset, and, in the long term, the prominent side effect is sexual dysfunction [15]. Higher rates of treatment adherence and fewer discontinuations have been reported with SSRIs relative to other antidepressant classes, making them one of the preferred first-line agents for pharmacologic treatment [14-16].

Following the introduction of the FDA black box warning, antidepressant prescriptions decreased by approximately 22% in the United States, while youth suicide rates increased by 14% the subsequent year - the largest year-to-year increase ever recorded by the CDC [17]. This pattern, combined with the underdiagnosis and inadequate treatment of pediatric MDD, may contribute to heightened vulnerability among children and adolescents.

In light of the black box warning, the FDA advises close monitoring of pediatric patients prescribed SSRIs for emerging suicidality [18]. Current professional guidelines from the American Academy of Child and Adolescent Psychiatry (AACAP) advise that pharmacotherapy with fluoxetine or escitalopram be the first-line treatment for moderate to severe depression, ideally in combination with psychotherapy [19]. The United Kingdom’s National Institute for Health and Care Excellence (NICE) guidelines dictate psychotherapy as the first-line treatment for mild to moderate pediatric depression, and SSRI use for severe or persistent depression only in combination with psychotherapy [20]. Overall, current global guidelines emphasize the role of SSRIs while cautioning close monitoring for treatment-emergent suicidality, structured follow-up, and multidisciplinary care. However, the findings remain inconsistent. A large population-based cohort study reported no increased risk of suicidal behavior associated with SSRI treatment and suggested that SSRIs may, in fact, reduce such risk [21]. Landmark clinical trials, such as the Treatment for Adolescents with Depression Study (TADS) and Treatment of SSRI-Resistant Depression in Adolescents (TORDIA), continue to emphasize the role of SSRIs in pediatric depression management [22,23]. These conflicting data highlight the need for continued investigation into the efficacy, safety, and tolerability of SSRIs as therapeutic options for pediatric and adolescent MDD.

## Review

Methods

An electronic literature search was conducted in PubMed in July 2024 without date restrictions. The search strategy combined the following Medical Subject Headings (MeSH) and text words to maximize sensitivity: “Selective Serotonin Reuptake Inhibitors”[Mesh], “Self-Injurious Behavior”[Mesh], “Adolescent”[Mesh] OR “Pediatrics”[Mesh] OR youth*[tw] OR teen*[tw] OR “young adult*”[tw] OR “young people”[tw] OR “high school”[tw]. To capture potentially relevant studies not indexed in PubMed, Google Scholar was also screened. 

The search yielded 371 records after deduplication. Titles were manually reviewed in the first screening stage, with abstracts consulted in cases of uncertainty regarding eligibility. As summarized in Table 1, irrelevant titles were excluded (n = 316), and the remaining studies were advanced for further screening.

**Table 1:** Selection strategy for articles SSRIs, Selective Serotonin Reuptake Inhibitors

Code	Status	Note
1	Excluded as irrelevant	Study title does not pertain to the use of SSRIs and the subsequent occurrence of suicidality in the target population
2	Subjected to screening using predefined inclusion and exclusion criteria	Pertains to the use of SSRIs and the subsequent occurrence of suicidality in the target population
3	Included for data extraction	Satisfies the below-mentioned inclusion and exclusion criteria

**Figure 1:**
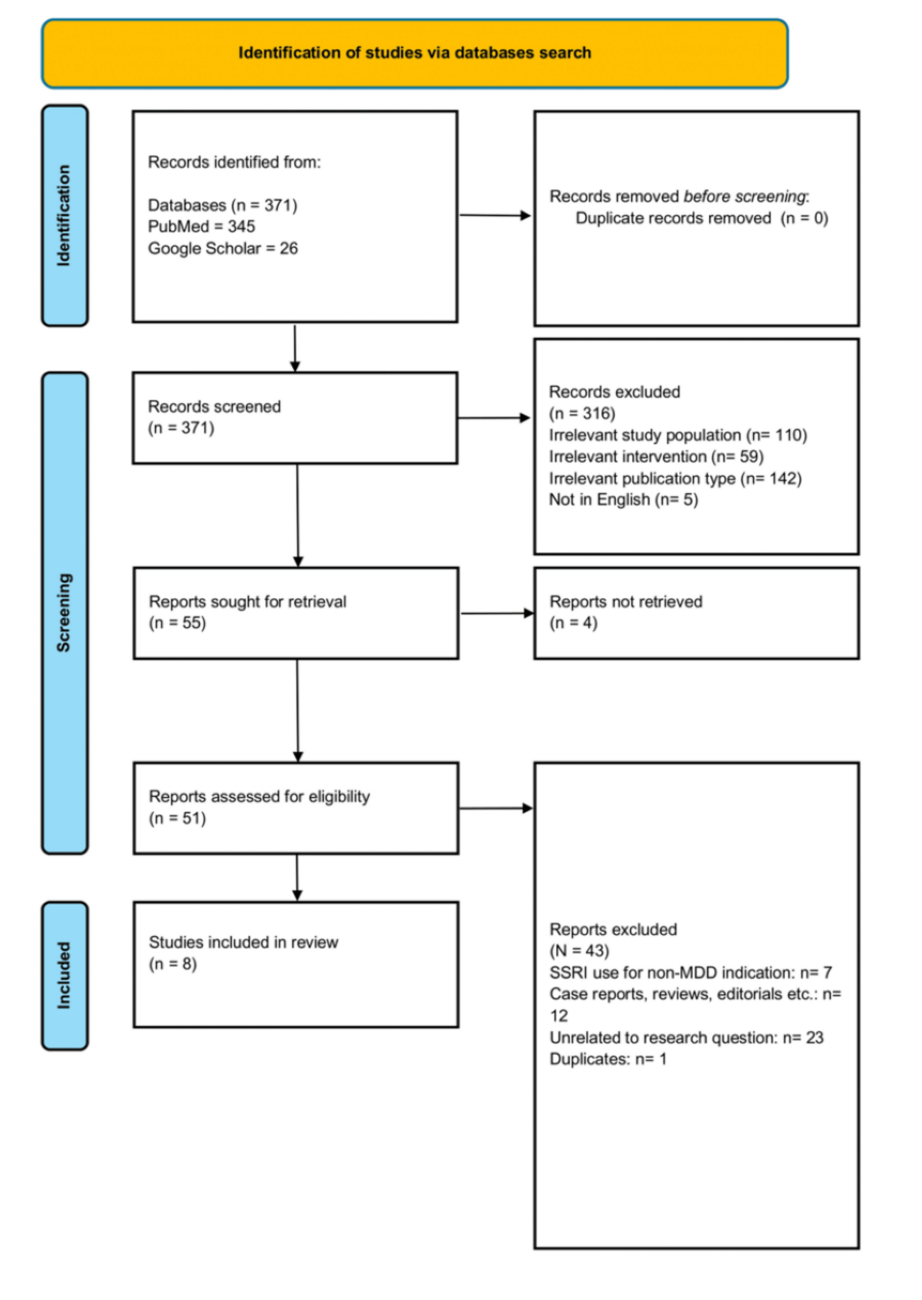
PRISMA 2020 flow diagram SSRIs, Selective Serotonin Reuptake Inhibitors; MDD, Major Depressive Disorder

As shown in Figure 1, the second-stage screening was performed on 51 of the 55 references identified, with four records deemed irretrievable. Eligibility was assessed according to three domains: study design, publication type, and study population. Included manuscripts were peer-reviewed publications in English of cross-sectional, case-control, cohort, randomized controlled trials (RCTs), and quasi-experimental designs that investigated the association between SSRIs use and suicidal behavior in the target population. Excluded study designs comprised case reports, narrative or systematic reviews, poster abstracts, unpublished dissertations, non-peer-reviewed publications, articles published in languages other than English, and studies that examined SSRIs' use in the treatment of psychiatric disorders other than MDD.

The target population consisted of patients younger than 18 years with a clinical diagnosis of MDD, preferably without additional psychiatric or medical comorbidities, who were undergoing treatment with SSRIs. 

Of the 51 publications screened, eight studies met the predefined inclusion and exclusion criteria and were retained for data extraction and analysis [22-29]. The remaining studies were excluded. Table 2 presents a summary of the included studies examining the association between SSRI use and suicidality in adolescents with MDD.

**Table 2:** Summary of included studies investigating the association between suicidality and SSRI use in adolescent MDD A-LIFE, Adolescent Longitudinal Interval Follow-Up Evaluation; AE, Adverse Events; ATD, Antidepressant(s); B.C., British Columbia; BSSRS, Brief Suicide Severity Rating Scale; C-CASA, Columbia Classification Algorithm of Suicide Assessment; CBT, Cognitive-Behavioral Therapy; CDRS-R, Children’s Depression Rating Scale-Revised; CGAS, Children’s Global Assessment Scale; CGI-I, Clinical Global Impressions-Improvement; COMB, Combination; DSM-IV, Diagnostic And Statistical Manual of Mental Disorders, Fourth Edition; FAERS, FDA Adverse Event Reporting System; FDA, Food And Drug Administration; FLX, Fluoxetine; ICD, International Classification of Diseases; MADRS, Montgomery-Asberg Depression Rating Scale; MDD, Major Depressive Disorder; MedDRA, Medical Dictionary For Regulatory Activities; NSRI, Non-Selective Monoamine Reuptake Inhibitors; NSSI, Non-Suicidal Self-Injury; OR, Odds Ratio; PBO, Placebo; QLESQ, Quality of Life Enjoyment And Satisfaction Questionnaire; RADS, Reynolds Adolescent Depression Scale; RCT, Randomized Control Trial; ROR, Reporting Odds Ratio; SA, Suicide Attempts; SAE, Suicidal Adverse Events; SB, Suicidal Behavior; SI, Suicidal Ideation; SNRI, Serotonin Norepinephrine Reuptake Inhibitor(s); SSRIs, Selective Serotonin Reuptake Inhibitors; TCA, Tricyclic Antidepressant(s); TORDIA, Treatment of Resistant Depression In Adolescents; U.K. GPRD, United Kingdom General Practice Research Database; VNF, Venlafaxine

Citation	Study Design	Objective to Assess	Population; N	Exposure	Outcome/Comparison	Results	Comments
March et al. (2007) and March et al. (2004) [22,24]	RCT conducted in the U.S.	Evaluate the effectiveness of FLX hydrochloride therapy, CBT, and their combination in adolescents with MDD over 12 weeks of treatment	N = 473; 12-17 year olds with a primary DSM-IV diagnosis of MDD	Random assignment to one of four groups: FLX, CBT, COMB, and PBO.	Response to treatment measured by CGI-I score. SI measured by CDRS-R. RADS measured the self-reported assessment of depressive symptoms. Harm-related AE were measured, which comprised SI, SA, and harm to others.	All active treatments were associated with significant reductions in suicidal thinking (p < 0.001), with combination therapy showing the greatest reduction. Treatment was most effective compared with placebo (p = 0.02) and CBT. The frequency of harm-related adverse events was higher in these groups (p > 0.02).	FLX is associated with an increase in harm-related AE, but reduced SI. PBO carried the highest risk of SA, suggesting that untreated depression carries more risk than SSRIs treatment. FLX is associated with more treatment-emergent side effects, but COMB treatment has the greatest symptom improvement and no SA. Clinically significant SI persists more commonly in FLX alone, but not in combination or CBT therapy, possibility for attribution bias.
Martinez et al. (2005) [25]	Nested case-control study conducted in the U.K.	Compare the risk of non-fatal self-harm and suicide in patients taking an SSRI or TCA for depression using data from the U.K. GPRD	N = 146,095; patients aged 10-64 years with first diagnosis of depression and ATD prescription	Current or recent use of a class of ATD into three groups: SSRIs, TCA, or other ATD use. TCA is the reference group.	Non-fatal self-harm comprised drug overdose, deliberate self-laceration, poisoning, and non-fatal suicide attempts using other methods. Cases of suicide are reported via medical codes and death certificates. Non-fatal self-harm and cases of suicide were compared across antidepressant types and age groups.	For adolescents, SSRIs may have a higher risk of non-fatal self-harm compared to TCA, OR 1.59 (1.01 to 2.50). All other age groups showed no evidence of increased risk. No suicides occurred in adolescent groups taking SSRIs or TCAs.	Confounding by indication: preferential prescribing of SSRIs to those with greater suicide risk.
Brent et al. (2009) [23]	RCT conducted in the U.S.	Identify predictors of self-harm AE in treatment-resistant depressed adolescents during the first 12 weeks of treatment	N = 334 ages 12-18 with treatment-resistant DSM-IV MDD	Two x two factorial RCT design with four groups: medication switch to another SSRIs, medication switch to VNF, switch to another SSRIs + CBT, switch to VNF + CBT	Voluntary reporting of suicidal and NSSI for the 1st 181 participants and systematic weekly assessment report for the last 153 participants. BSSRS has two components: SI scale and SB scale. Only the SB scale was classified by using C-CASA. Self-harm events were classified by raters who were blinded to medication but not CBT assignment.	No statistically significant treatment effects for the occurrence of SAE and NSSI in any group. Participants with higher than median baseline SI were more likely to experience SAE with VNF than with an SSRIs (p = 0.05).	SSRIs' use is less risky than VNF use in high-risk adolescents. CBT failed to attenuate the risk of NSSI but is linked to earlier NSSI detection.
Schneeweiss et al. (2010) [26]	Cohort study conducted in B.C.	To assess the risk of SA and suicides after initiating ATD	N = 20,906; ages 10-18 years with diagnosed depression; 16,774 (80%) has no previous ATD use	Initiation of ATD agents: SSRIs, SNRIs, TCAs, and others. Within the SSRIs, FLX was the reference group.	Occurrence of SA was defined as a hospitalization with an ICD-9, and an E-code of E950.x–E958.x for deliberate self-harm. The occurrence of completed suicide was defined as deaths with an ICD-9 code E-code of E950.x to E958.x, or ICD-10 code of X60 to X84.	No statistically significant differences among SSRIs or between SSRIs and other classes.	Findings support the FDA's decision to include a black box warning on all ATD for adolescent MDD. SSRIs' use does not cause more suicidality than other ATDs.
Vitiello et al. (2011) [27]	Observational follow-up of the TORDIA RCT conducted in the U.S.	Observe the long-term effects of the TORDIA trial population over 72 weeks	N = 334 ages 12-18 with treatment-resistant DSM-IV MDD	Two by two factorial RCT design with four groups: medication switch to another SSRIs, medication switch to VNF, switch to another SSRIs + CBT, switch to VNF + CBT	CDRS-R and CGI-I are used to measure treatment response. A-LIFE tool measured remission and relapse rates. CGAS measured the functional recovery rate.	Remission rate and time to remission are not statistically significant. The SSRI group had a more rapid decline in self-reported depressive symptoms and suicidal ideation (SI) than the VNF group (p < 0.05).	SSRIs-treated adolescents are less suicidal more quickly than VNF.
Umetsu et al. (2015) [28]	Case-control retrospective study using FAERS	Evaluate the association between SSRIs therapy and suicidality	N = 2695; ages under 18 with MDD. Data sub-setting from a larger study population	Exposure to SSRIs use and NSRI use, mainly TCAs	Suicidal and self-harm events were reported to FAERS, identified using MedDRA preferred terms, and calculated as ROR. The depression subset group was used to control by indication.	Suicidal events, adjusted for the whole dataset, had an ROR of 9.58 (p < 0.0001). In the subset, ROR = 4.64 (p < 0.0001). Self-harm events, adjusted for the whole dataset, had an ROR of 31.40 (p < 0.0001). In the subset, ROR = 16.31 (p < 0.0003).	Data sub-setting revealed that the association between SSRIs and suicidality is likely smaller than previously understood. Although adjusted RORs were lower in subset analyses, there is an association between SSRIs treatment and suicidal and self-harm events, and these associations were stronger in the adolescent group than in other age groups.
Davey et al. (2019) [29]	RCT conducted in Australia	Evaluate whether FLX provides benefit over CBT alone in adolescents	N = 153; ages 15-25 years with MDD	Randomization to two groups: FLX + CBT, PBO + CBT	MADRS measured depressive symptoms. QLESQ measured self-reported quality of life. Adverse events coded using MedDRA.	No significant difference in the reduction of depressive symptoms, quality of life, and AE.	Findings do not support routinely adding FLX to treat adolescent MDD, in contrast to the TADS study findings.

Discussion

To our knowledge, a few recent reviews have specifically evaluated the association between SSRI use for MDD in patients under 18 years of age and subsequent suicidality. Existing reviews often examine suicidality associated with SSRI use in other psychiatric conditions (e.g., pediatric non-obsessive compulsive anxiety disorders [30]) or in different age groups (e.g., geriatric MDD [31]). A 2021 network meta-analysis of newer-generation antidepressants in children and adolescents by Hetrick et al. synthesized average effects across multiple agents rather than focusing on individual drugs [32]. Addressing this gap, the present systematized review [9] provides a focused synthesis of SSRIs in pediatric MDD, aiming to guide clinicians in weighing therapeutic benefits against potential risks of suicidality [9]. Eight studies [22-29] met eligibility criteria and were included for data extraction and synthesis. Of these, four were observational studies [25-28], and four were RCTs [22-24,29].

Three observational studies [25,26,28] emphasized the need for vigilant monitoring of adolescents treated with SSRIs. Martinez et al. [25], in a nested case-control study, found evidence of an increased risk of nonfatal self-harm with SSRIs compared to TCAs among adolescents, although this association may be confounded by indication, as SSRIs are more frequently prescribed to patients at higher baseline risk. Umetsu et al. [28], in a large retrospective case-control study, reported an elevated association between SSRI use and suicidal events in adolescents relative to other age groups, though subgroup analyses suggested that the effect size may be smaller than previously assumed. Schneeweiss et al. [26], in a cohort study, observed no statistically significant difference in suicide attempts or completed suicides between SSRIs, TCAs, SNRIs, and other antidepressants. Nevertheless, they recommended a black box warning for all antidepressant classes in adolescent MDD. By contrast, Vitiello et al. [27], in a 72-week follow-up study of the TORDIA cohort, found SSRIs associated with more rapid improvement in depressive symptoms and suicidal ideation compared to venlafaxine, supporting their safety and efficacy.

Two RCTs found that combined treatment with SSRIs and CBT was associated with the greatest symptom improvement and the largest reduction in suicidal ideation [22,24]. March et al. [22] reported higher rates of treatment-emergent suicidality with fluoxetine monotherapy compared to CBT or combined treatment, highlighting CBT’s potential protective role. In contrast, two other RCTs reported no significant increase in suicidal adverse events with SSRIs [23,29]. Brent et al. [23] randomized adolescents with treatment-resistant depression to four groups (SSRIs, venlafaxine, SSRIs + CBT, and venlafaxine + CBT) and found no significant difference in suicidality across groups. Notably, participants with high baseline suicidal ideation experienced more self-harm with venlafaxine than with SSRIs. CBT did not reduce the incidence of nonsuicidal self-injury but facilitated earlier detection. Davey et al. [29] found no difference in depressive symptom reduction or suicidality between fluoxetine + CBT and placebo + CBT groups, thus not supporting routine fluoxetine augmentation.

Overall, two RCTs suggested an increased risk of suicidality with SSRI monotherapy [22,24] but demonstrated protective effects of combined CBT, while two RCTs found no increased risk [23,29]. Observational studies showed mixed findings: most studies reported some degree of elevated risk [25,26,28], though one study supported SSRI safety [27]. Collectively, this body of evidence suggests that SSRI use in adolescents with MDD may be associated with a small but measurable increase in suicidality, particularly early in treatment, reinforcing the importance of close monitoring and integration of psychotherapy. A meta-analysis of 34 RCTs conducted through 2015 reported fluoxetine to be superior to placebo and more tolerable than duloxetine or imipramine, as assessed by discontinuation rates [33]. Another systematic review of eight observational studies, including >200,000 patients, found increased suicidality risk with SSRIs only in depressed adolescents, not in other age groups [34]. These findings align with the present review.

Strengths and limitations

This review provides an updated synthesis of the evidence following the 2004 FDA black box warning, focusing on a high-risk population often underrepresented in prior reviews. Systematized reviews are well-suited for fragmented evidence bases, consolidating heterogeneous findings into a clinically relevant overview [9]. However, limitations include the absence of a formal quality assessment, heterogeneity in study design (with RCTs generally reporting no or reduced risk, and observational studies showing elevated risk), and the conduct of screening, extraction, and synthesis by a single reviewer, which introduces potential selection bias.

## Conclusions

This systematized literature review found a measurable association between SSRI use for the treatment of adolescent major MDD and an increased risk of suicidality. Observational studies consistently demonstrated a heightened risk, whereas findings from RCTs were mixed. While some RCTs reported increased suicidal ideation with SSRI monotherapy, most found no significant association between SSRI use and suicidality. Notably, CBT emerged as a protective factor, reducing treatment-emergent suicidality and facilitating earlier detection of NSSI during treatment. Overall, although SSRIs remain a key therapeutic option for adolescent MDD, these findings underscore the importance of close suicidality monitoring during the early treatment phase and support the integration of CBT as an adjunctive intervention. Given the ethical challenges associated with researching suicidality and the mixed findings across studies included in this review, clinicians must rely on clinical experience and anecdotal evidence when considering the use of SSRIs for treating adolescent MDD. This consideration is critical, as untreated depression itself remains a significant risk factor for suicide.
